# Long noncoding RNA MALAT-1 is a novel inflammatory regulator in human systemic lupus erythematosus

**DOI:** 10.18632/oncotarget.20490

**Published:** 2017-08-24

**Authors:** Huaxia Yang, Naixin Liang, Min Wang, Yunyun Fei, Jian Sun, Zhiyuan Li, Yuan Xu, Chao Guo, Zhili Cao, Shanqing Li, Yuchen Jiao

**Affiliations:** ^1^ Department of Rheumatology and Clinical Immunology, Clinical Immunology Center, The Ministry of Education Key Laboratory, Peking Union Medical College Hospital, Chinese Academy of Medical Sciences and Peking Union Medical College, Beijing, China; ^2^ Department of Thoracic Surgery, Peking Union Medical College Hospital, Peking Union Medical College and Chinese Academy of Medical Sciences, Beijing, China; ^3^ Department of Pathology, Peking Union Medical College Hospital, Peking Union Medical College and Chinese Academy of Medical Sciences, Beijing, China; ^4^ National Key Laboratory of Molecular Oncology, Chinese Academy of Medical Sciences Cancer Hospital, Beijing, China

**Keywords:** MALAT-1, lncRNA, SLE, PBMC, IL-21

## Abstract

Despite growing evidence that Long noncoding RNAs (lncRNAs) can regulate gene expression and widely take part in autoimmune and inflammatory diseases, our knowledge of systemic lupus erythematosus (SLE)-related lincRNAs remains limited. In this study, we aimed to explore the contribution of the lncRNA metastasis associated lung adenocarcinoma transcript 1 (MALAT1) to the pathogenesis of SLE. PBMCs were obtained from SLE patients and healthy donors. The expression levels of MALAT-1 were measured by quantitative PCR. Small interfering RNA (siRNA) was then used to knock down the expression of MALAT1 in order to determine the role of MALAT1 in the expression levels of IL-21 and SIRT1 signaling pathway in primary monocytes of SLE patients. Here, we found MALAT-1 expression was abnormally increased in SLE patients and predominantly expressed in human monocytes. Additionally, silencing MALAT-1 significantly reduced the expression of IL-21 in primary monocytes of SLE patients. Furthermore, MALAT-1 exerts its detrimental effects by regulating SIRT1 signaling. Our results demonstrate that MALAT-1 is the key regulatory factor in the pathogenesis of SLE and provides potentially novel target for therapeutic intervention.

## INTRODUCTION

Systemic lupus erythematosus (SLE), a heterogeneous chronic inflammatory autoimmune disorder characterized by inflammation in multiple organ systems, causes serious injury to various organs or systems [1, 2]. The accumulated dying cells release autoantigens further breaking down the immune tolerance of T and B cells and triggering SLE [3]. Although molecular characteristics of SLE have been investigated, the crucial contributor to the pathogenesis of SLE remains poorly understood.

Long noncoding RNAs (lncRNAs) are defined as ncRNAs that longer than 200 nucleotides without the capacity of coding proteins, located within the intergenic stretches or overlapping antisense transcripts of protein coding genes [4]. Anomalous expressions of lncRNAs are associated with various human diseases [5]. There is growing evidence that lncRNAs participate in various biological events, and recent studies have suggested that dysregulation of lncRNAs are involved in the pathogenesis of autoimmune and inflammatory diseases [6]. However, only a few lncRNAs have been indicated in the SLE-associated aberrant gene networks [7]. LncRNAs are differentially expressed in peripheral blood mononuclear cells (PBMCs), immortalized B cells, and kidney biopsy specimens from SLE patients [8].

Both the aforementioned studies have suggested that the metastasis-associated lung adenocarcinoma transcript 1 (MALAT-1) is upregulated in several cancer tissues [9] and it might be involved in both the process of tumorigenesis as well as metastatic progression [10]. However, the overall biologic role and the underlying molecular mechanism of MALAT-1 in SLE biologyremain largely undefined.

## RESULTS

### MALAT-1 lncRNA expression is upregulated in SLE patients

**Figure 1:**
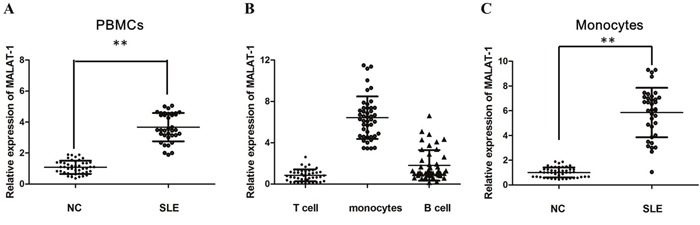
The relative expression of MALAT-1 was determined by qPCR in SLE patients **(A)** Expression of MALAT-1 in PBMCs of SLE patients and normal controls (NC), as determined by qPCR analysis. **(B)** Expression of MALAT-1 in T cells, monocytes and B cells from healthy donors. **(C)** MALAT-1 expression was up-regulated in monocytes of SLE patients compared with normal controls. ** P < 0.01.

To determine MALAT-1 that may contribute to SLE, the level of MALAT-1 expression was determined in PBMCs obtained from SLE patients and Healthy controls by qRT-PCR analysis. Increased MALAT-1 expression was detected in the PBMCs from SLE patients compared with that from normal controls (Figure 1A). To determine the cellular-specificity of MALAT-1 expression, we examined the expression of MALAT-1 in the main subsets of PBMC (T cells, monocytes and B cells) from healthy donors. MALAT-1 was expressed at substantially increased levels in monocytes compared with T and B cells (Figure 1B). In addition, separated CD14-positive monocytes from PBMC of SLE patients showed notably enriched MALAT-1 expression as compared with normal controls (Figure 1C), implying that the monocyte is the primary MALAT-expressing cell type.

### MALAT-1 increases IL-21 in primary monocytes of SLE patients

**Figure 2:**
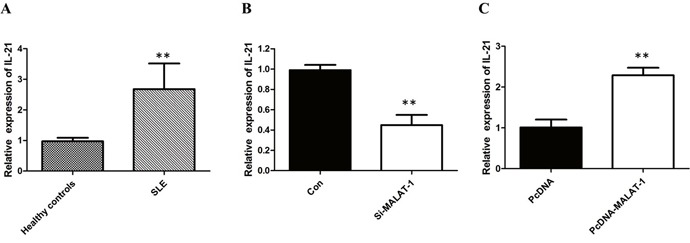
MALAT-1 regulates the expression of IL-21 **(A)** The results of qRT-PCR analysis showed that the mRNA of IL-21 was significantly increased in human primary monocytes of SLE groups. **(B)** Knockdown of MALAT-1 significantly down-regulated the mRNA level of IL-21. **(C)** Overexpression of MALAT-1 significantly increased the mRNA level of IL-21. ** P < 0.01.

Monocyte-mediated cytokines plays an important role in the pathogenesis of SLE, in which the level of IL-21 is a key factor for disease activity. The results of qRT-PCR analysis showed that the mRNA of IL-21 was significantly increased in human primary monocytes of SLE groups (*P* < 0.01; Figure 2A).

**Figure 3:**
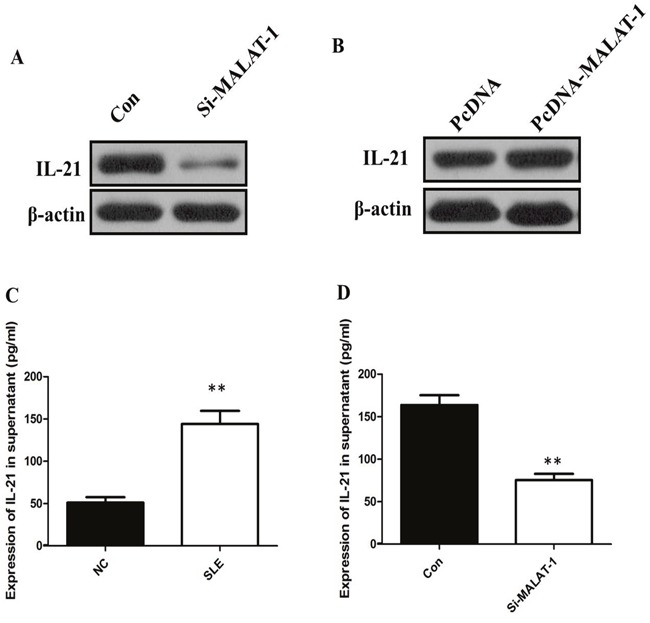
**(A)** Western blot analysis showed that inhibition of MALAT-1 significantly down-regulated the protein level of IL-21. **(B)** Overexpression of MALAT-1 increased the IL-21 protein. **(C)** ELISA analysis showed that the IL-21 level in supernatant of SLE group was increased more than three times than that in Healthy control. **(D)** Inhibition of MALAT-1 decreased the IL-10 level in supernatant. ** P < 0.01.

To investigate whether the level of IL-21 in monocytes from SLE patients is associated with MALAT-1, monocytes were infected with the MALAT-1 siRNA. Conversely, for gain of function studies, a pcDNA-MALAT-1 vector was transiently transfected to ectopically overexpress MALAT-1 in monocytes. Knockdown of MALAT-1 significantly down-regulated the mRNA level of IL-21, while MALAT-1overexpression significantly enhanced the expression of IL-21 in monocytes (Figure 2B and 2C). Western blot analysis showed that MALAT-1 knockdown significantly down-regulated the protein level of IL-21, and overexpression of MALAT-1 increased the IL-21 protein (Figure 3A and 3B).

The presence of the proinflammatory cytokines IL-21 between Healthy control groups and SLE groups was determined by ELISA. Our data showed that the IL-21 level in supernatant of SLE group was increased more than three times than that in Healthy control (*P* < 0.01, Figure 3C). In addition, the IL-21 levels in the culture media of monocytes that were untreated controls and IL-21 transfected with the specific siRNA of MALAT-1. When compared with the control group, the IL-21levels in the culture medium were reduced significantly after inhibition of MALAT-1 (*P* < 0.01, Figure 3D). Together, these results support the hypothesis that MALAT-1 expression plays an important role in the expression levels of IL-10 in monocytes.

### MALAT-1 exerts its detrimental effects by regulating SIRT1 signaling

**Figure 4:**
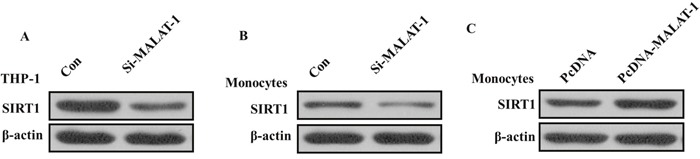
**(A)** Western blot analysis showed that the expression of SIRT1 was significantly decreased after knockdown of MALAT1 in THP-1 cells. **(B)** Western blot analysis showed that knockdown of MALAT-1 could significantly down-regulated the expression of SIRT1 in monocytes. **(C)** Western blot analysis showed that overexpression of MALAT-1 could induce the expression of SIRT1 in monocytes.

Previous studies showed that MALAT1is involved in regulation of SIRT1 signaling that contributed to apoptosis and reversion of activated LX-2 cells in liver fibrosis [11]. As emerging evidence reported that SIRT1 contributed to the initiation and maintenance of lupus disease, we wondered that whether MALAT1 related to increased expression of SIRT1 in monocytes from SLE patients. Considering the expression level of SIRT1 in THP-1 human monocytic cell line [12], we stimulated the THP-1 human monocytic cell line which has been used extensively to study the innate immune response with various innate immunity ligands [13], and found that the expression of SIRT1 was significantly decreased in THP-1 cells after MALAT1 knockdown (Figure 4A). We also found that knockdown of MALAT-1 could significantly down-regulated the expression of SIRT1, and overexpression of MALAT-1 could induce the expression of SIRT1 in human primary monocytes freshly isolated from PBMCs (Figure 4B and 4C), suggesting that MALAT-1 exerts its detrimental effects by regulating SIRT1 signaling in both THP-1 cell lines and human primary monocytes.

## DISCUSSION

SLE affects multiple systems and organs by multiple autoantibodies [14]. SLE is featured by insidious or abrupt onset with severe and relapsing course, [15]. The development of SLE involves the disorders of nearly the entire immune system [16]. Despite great advances in modern medicine, the treatment of SLE still remains difficult, especially in its earlier stages. Therefore, to clearly elucidate the mechanism of SLE progression and to design an effective therapeutic strategy to treatment of SLE is urgently needed.

Recent studies have shown that lncRNAs play important roles in the disorder of immune system including SLE [7, 17]. MALAT1 is an abundantly expressed nuclear lncRNA measuring approximately 8000 nucleotides in length [18]. MALAT1, located on chromosome 11 (11q13.1), is one of the few biologically well-studied lncRNAs [19]. With respect to its function, MALAT1 is localized to nuclear speckles and has been associated with regulation of gene expressions [20]. The role and function of MALAT1 has not yet been annotated in exact etiology of SLE.

In this study, we elucidated the role of MALAT1 in the pathogenesis of SLE by investigate the expression of MALAT1 in Chinese SLE patients and healthy controls. We found that levels of MALAT1 were increased in PBMCs from SLE patients comparing to that in healthy controls. Monocytes were one of the major components of innate immune system. In recent years, monocytes have been found to severely altered in phenotype and lineage flexibility in SLE patients. We found MALAT-1 was also significantly up-regulated in monocytes of SLE patients as compared with normal controls. As secretion inflammatory cytokines in response to innate immunity ligands is one of the prominent features of monocytes. Moreover, the production level of IL-21 was increased in monocytes of SLE patients whereas inhibition of MALAT-1 decreased the IL-21 level in monocytes. Also, we found that MALAT-1 regulated the SIRT1 pathway directly in monocytes of SLE patients. Silence of MALAT-1 decreased the level of SIRT1 by Western blot analysis in monocytes of SLE patients. Overexpression of MALAT-1 exerted effects that were diametrically opposed to those observed with MALAT-1 knockdown, indicating that MALAT-1 plays an important role in the pathogenesis and development of SLE through SIRT1signaling pathway.

In conclusion, we demonstrate for the first time that MALAT-1 is the key regulatory factor in the pathogenesis of SLE, which regulated the expression of IL-21 and SIRT1 in monocytes of SLE patients. These data suggest a novel function and a therapeutic application of MALAT-1 in SLE.

## PATIENTS AND METHODS

### Study subjects

36 patients with SLE and 45 age-matched and sex-matched normal controls were recruited for isolating PBMCs and then sorting monocytes by flow cytometry and testing expression of MALAT-1 in both PBMCs and monocytes. Healthy donors had no history of autoimmune diseases or treatment with immunosuppressive agents. Patients with concurrent infection were excluded from the study. They had never been treated with disease-modifying antirheumatic drugs or other immunosuppressive drugs. All SLE patients fulfilled the American College of Rheumatology (ACR) classification criteria for SLE. The Systemic Lupus Erythematosus Disease Activity Index (SLEDAI) score was determined for each patient at the time of the blood draw. Patients were categorized as having active disease (scores >4) or inactive disease (scores ≤4) based on the SLEDAI results. The study was approved by the Research Ethics Board of Peking Union Medical College Hospital. Informed consent was obtained from all study participants.

### Isolation of peripheral blood mononuclear cells (PBMCs), T cells, B cells and monocytes

Isolation of PBMCs, T cells, B cells and monocytes Whole blood (10 ml) was collected in EDTA collection tubes from each subject, and PBMCs were isolated by density-gradient centrifugation with Ficoll-Paque Premium (GE Healthcate), according to the instructions. For the subsets of PBMCs isolation, the fresh PBMCs were incubated for 15 min at 4°C with flurescentconjugated monoclonal antibodies: anti-CD3-PerCP-Cy5.5, anti- CD14-PE, anti-CD19-APC (all from BD Biosciences). Stained cells were sorted on a BD FACSAria III (BD Biosciences). T cells were identified as CD3t/CD19-. Monocytes were isolated if cells were CD14t/CD3-. B cells were collected if cells were CD19t/CD3-. The stained cells were sorted to >98% purity.

### Cell culture and transfection

The THP-1 cells were obtained from the Cell Bank, Shanghai Institutes for Biological Sciences. THP-1 cells were grown in RPMI 1640 (Gibco, Life technology) with 10% fetal bovine serum (Gibco, Life technology) at 37°C in a 5% CO_2_ atmosphere. Cell lines were transfected using Lipofectamine 2000 (Invitrogen). PBMCs were isolated using a density gradient separation medium (Cedarlane, Burlington, NC) and were rested in RPMI 1640 supplemented with 10% FBS for 2 hours. Two sets of short hairpin RNA (shRNA) targeting 5’-GGCTCTTCCTTCTGTTCTA-3’ (6427-6445) and 5’-GAAGGAGCTTCCAGTTGAA-3’ (7211-7229) on MALAT-1 transcript were cloned into the pRNA-H1.1/Neo siRNA expression vector (GenScript, Piscataway, NJ, USA) to make MALAT-1 shRNA-1 and MALAT-1 shRNA-2 respectively. The scramble shRNA with the sequence of 5’-TTCTCCGAACGTGTCACGTTTCAAGAGAACGTGACACGTTCGGAGAA-3’ was cloned into pRNA-H1.1/Neo as the negative control (NC). Cells were transfected with the indicated vector using lipofectamin 2000 (Invitrogen, Carlsbad, CA, USA) according to the manufacturer's instructions. Prior to transfection, the cells were starved in serum-free medium for 1 h. The medium was replaced with fresh culture medium 6 h after transfection.

### RNA was extracted by Trizol reagent method

Total RNA was extracted using TRIzol (Invitrogen, San Diego, CA). Approximately 1 μg of RNA was reverse transcribed into complementary DNA (cDNA) using Superscript II reverse transcriptase (Invitrogen) and oligo(dT) primers. Reverse transcription in 20 μL system was preformed following protocol of Applied Biosystems. Primers for RT-qPCR were MALAT-1: F-GAATTGCGTCATTTAAAGCCTAGTT, R-GTTTCATCCTACCACTCCCAATTAAT; GAPDH: F-ACAGTCAGCCGCATCTTCTT, R-GACAAGCTTC CCGTTCTCAG. Quantitative mRNA expression was measured by ViiA^™^ 7 Real-Time PCR System (Applied Biosystems Inc., Foster City, CA, USA). The expression of GAPDH was detected as the endogenous control. Relative mRNA expression of MALAT-1 was calculated with the comparative threshold cycle (C_t_) (2^−ΔΔCt^) method.

### Enzyme-linked immunosorbent assay (ELISA)

The amount of IL-21 proteins secreted into the cell culture supernatant was quantified using enzyme-linked immunosorbent assay (ELISA) kits (Neobioscience Bioengineering Co., Shenzhen, China) according to the manufacturer's instructions and the absorbance of the samples were read at 450 nm using a microplate reader (Biotek, USA).

### Western blot

Total proteins were extracted from cells or tissues using RIPA buffer (50 mM Tris-HCl, pH 7.4, 150 mM NaCl, 1 mM EDTA, 1% Triton X-100, 1% sodium deoxycholate, 0.1% SDS) supplemented with 1 mM PMSF, 5 μg/ml aprotinin, 5 μg/ml leupeptin. The protein concentration was determined by BCA Protein Assay Kit (Vigorous, China). A total of 20 μg protein was loaded to 10% SDS-PAGE gel, probed with primary antibodies again IL-21,SIRT1 and GAPDH (Abcam) followed by horseradish peroxidase-(HRP) conjugated sheep anti-mouse or rabbit Ig (ZSGB-BIO).

### Statistics

Student's t-test (two-tailed) was performed to analyze the data. A two-sided P-value of less than 0.05 was considered statistically significant. All statistical computations were performed using SPSS (SPSS Inc., USA).
